# Supplementary material for: Characterization of Two Glycoside Hydrolases of Family GH13 and GH57, Present in a Polysaccharide Utilization Locus (PUL) of Pontibacter sp. SGAir0037
Source: Molecules. 2024 Jun 12;29(12):2788. doi: 10.3390/molecules29122788 (PMC11206854; doi:10.3390/molecules29122788)
Supplement: Supplementary file 1 [file molecules-29-02788-s001.zip › molecules-3048190-supplementary.pdf]

Supplementary data

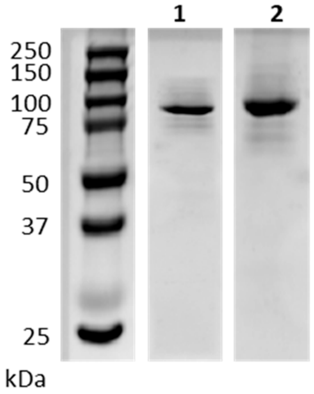

Figure S1. SDS-PAGE gel of purified PoGBE13 (*Pontibacter* sp. SGAir0037; 92 kDa, lane 1) and PoGH57 (*Pontibacter* sp. SGAir0037, 93 kDa, lane 2). Protein amount on gel is 3 µg.

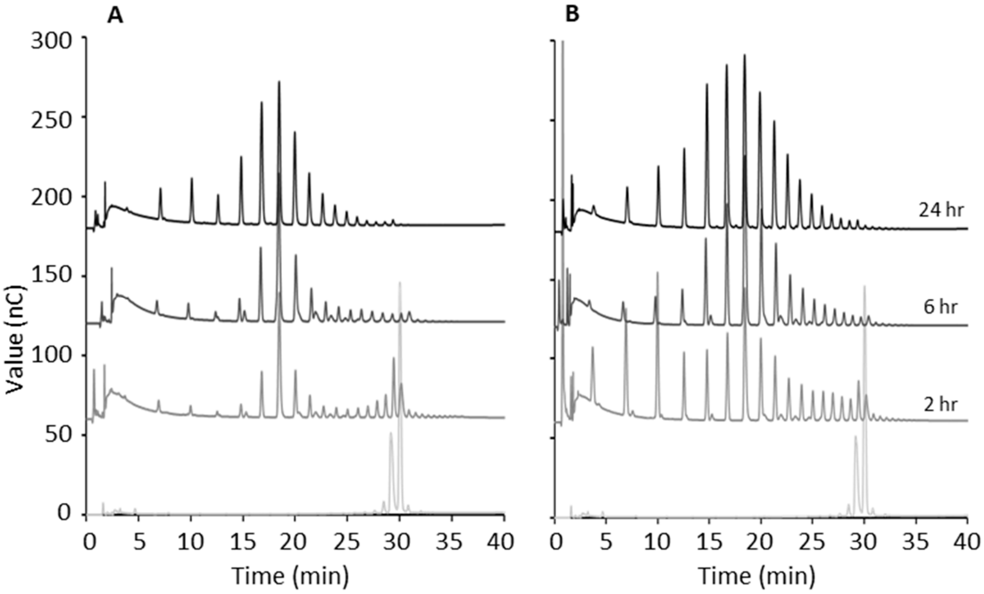

Figure S2. Chain length distribution of MD18 modified with PoGBE13 (*Pontibacter* sp. SGAir0037; 1U[B]/g S) over time before [A] and after [B] debranching compared to the untreated substrate.

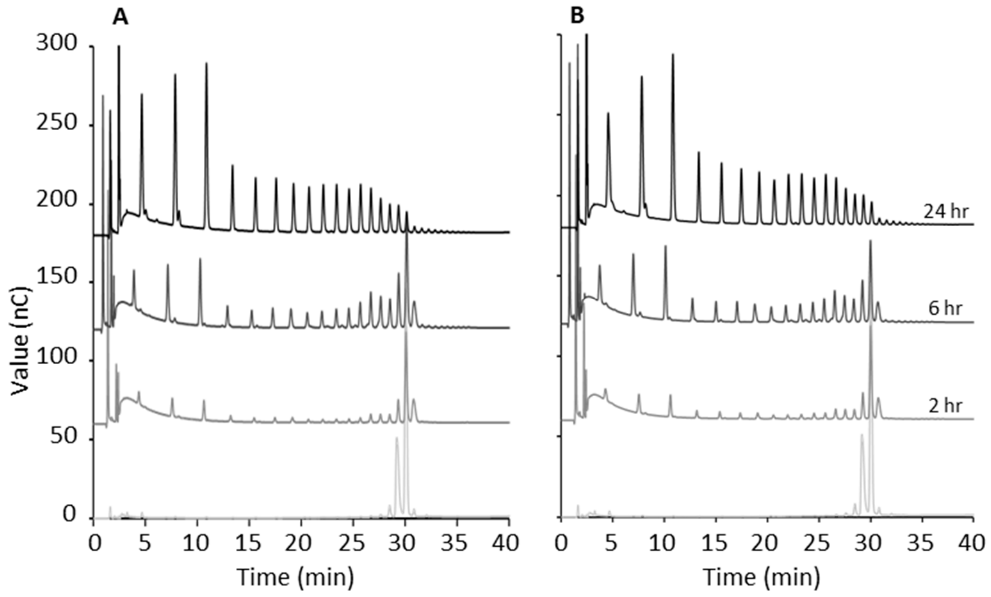

Figure S3. Chain length distribution of MD18 modified with PoGH57 (*Pontibacter* sp. SGAir0037*;* 1U^B/g S) over time before [A] and after [B] debranching compared to the untreated substrate.

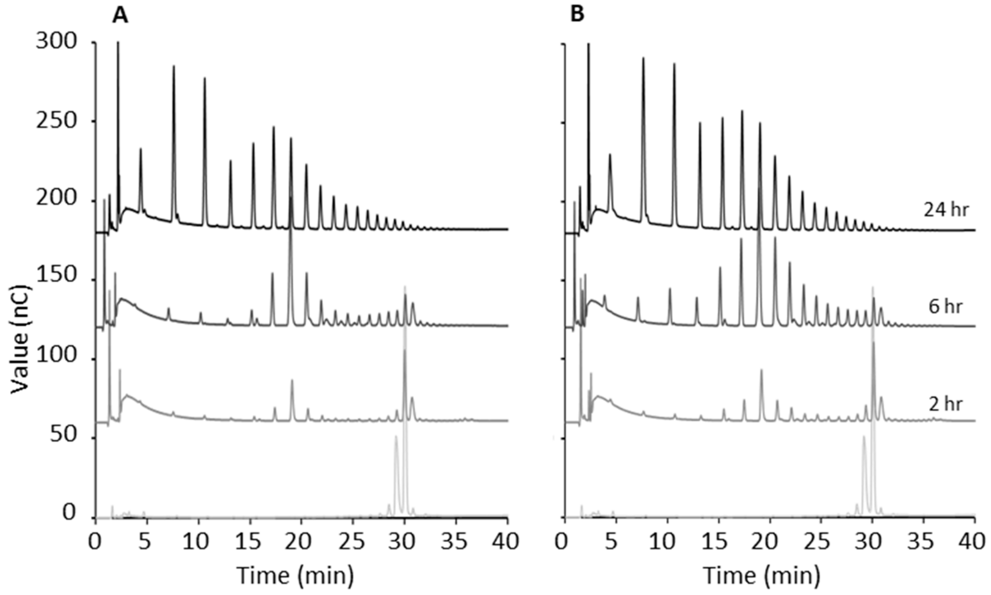

Figure S4. Chain length distribution of MD18 modified with PoGBE13 & PoGH57 (*Pontibacter* sp. SGAir0037*;* 1U^B/g S) in a one-pot reaction over time before [A] and after [B] debranching compared to the untreated substrate.
